# Improvement in protein HSQC spectra from addition of betaine

**DOI:** 10.1007/s10858-025-00463-0

**Published:** 2025-03-10

**Authors:** Finn O’Dea, Aiden J. Seargeant, Jessica Hurcum, Rodolpho do Aido-Machado, Michelle L. Rowe, Nicola J. Baxter, Jon P. Waltho, Jon R. Sayers, Mike P. Williamson

**Affiliations:** 1 School of Biosciences, University of Sheffield, Sheffield, S10 2TN UK; 2 Division of Clinical Medicine, School of Medicine and Population Health, University of Sheffield, Sheffield, S10 2RX UK

**Keywords:** Osmolyte, Betaine, Fluctuation, Dynamics, Amide exchange, Barnase, Flap endonuclease

## Abstract

Addition of glycine betaine up to 1 M gave rise to increased intensity for some weak signals in the HSQC spectra of barnase and *Plasmodium falciparum* flap endonuclease. The signals that were enhanced were low intensity signals, often from amide groups with rapid internal motion (low order parameter). The majority of signals are however somewhat weaker because of the increased viscosity. Addition of betaine is shown to cause a small but significant overall increase in order parameter, no consistent effect on conformational change on the µs-ms timescale, and a reduction in amide exchange rates, by a factor of about 3. The results are consistent with a model in which betaine leads to a reduction in fluctuations within the bulk water, which in turn produces a reduction in internal fluctuations of the protein.

## Introduction

Osmolytes are small organic molecules produced by many organisms and accumulated to high concentration inside the cell, as a response to stresses such as high osmotic pressure, high temperature, extremes of pH, or high hydrostatic pressure (Yancey 2005). They stabilise proteins and help to keep the cell functioning. They have a range of chemical structures, including amino acids and their derivatives (proline, glycine, ectoine), polyols (trehalose, glycerol and many sugar derivatives), and methylamines (trimethylamine N-oxide [TMAO] and N-trimethylglycine [betaine]). They are highly soluble in water and bind more strongly to water than to protein surfaces, as a result of which they are said to be excluded from the protein surface: in other words, the concentration of osmolyte at the protein surface is lower than it is in bulk water (Cayley and Record 2003). For this reason, they are also described as compatible solutes. The exception to this rule is urea, which is also an osmolyte, but binds to proteins and destabilises them.

Because of their ability to stabilise proteins in solution, osmolytes have been much studied. We recently conducted a study looking at the effects of osmolytes and Hofmeister ions on the protein barnase (Trevitt et al. 2024), during the course of which we noticed that some signal intensities seemed to increase with addition of osmolytes. We therefore felt that this was an observation worth pursuing. Here, we report on studies with two different proteins and show that in both cases, addition of betaine causes an increase in the intensity of a small group of signals. We provide an explanation for the mechanistic origin of the effect, which we suggest is due to the ability of betaine to reduce fluctuations in the bulk water.

## Materials and methods

Barnase H102A was expressed in *Escherichia coli* M15 [pREP4] cells from a pQE60 plasmid, in M9 media containing 1 g/l (^15^NH_4_)_2_SO_4_, and purified using ion exchange chromatography as described (Cioffi et al. 2009). Flap endonuclease (FEN) from *P. falciparum* was expressed in *E. coli* cells and purified using ammonium sulphate precipitation, followed by ion exchange, heparin and gel filtration chromatography as described (do Aido-Machado et al. 2025). Purities of each protein were checked by SDS-PAGE and confirmed by preliminary HSQC spectra. The buffers for barnase and FEN were 5 mM sodium acetate pH 5.8; and 20 mM sodium phosphate pH 7.4, 30 mM KCl, 10 mM MgCl_2_, 0.1 mM EDTA, 2 mM Tris(2-carboxyethyl) phosphine, respectively. All buffers also contained sodium azide and sodium trimethylsilypropionate (TSP).

For each betaine titration, a solution of 3 M betaine was prepared in the appropriate buffer, and the pH was adjusted. Solutions were prepared by mixing appropriate volumes of betaine, protein and buffer, with the addition of 10% D_2_O.

NMR measurements were carried out on a Bruker 600 MHz Neo (barnase) or 800 MHz Neo (FEN). Spectra were processed using Topspin 4.0.5 and analysed in Felix (Felix NMR Inc, San Diego, CA) to obtain chemical shifts and intensities. Spectra were processed identically using a cosine-squared bell in both dimensions and no baseline correction. For analysis of signal intensity as a function of betaine concentration, measured intensities were normalised so that the majority of backbone and sidechain signals retained an approximately constant intensity throughout. Amide exchange was carried out by lyophilising a solution of protein in H_2_O plus buffer, and then adding the same volume of D_2_O. Sample temperature was 30 °C, in 50 mM tris, pH 6.7. Measured intensities were corrected for protein precipitation and for the presence of 2.5% residual protons in the D_2_O. Relaxation data were measured using a Bruker temperature-compensated pulse program. For relaxation analysis of barnase, we used a sample grown in D_2_O and subsequently back-exchanged into H_2_O, so that amides are close to 100% ^1^H, but all other hydrogen atoms are approximately 97% ^2^H, to limit dipolar relaxation to directly bonded ^1^H-^15^N. The *T*_1_ experiments used 10 inversion delays from 10 ms to 1200 ms, including two duplicates; and the *T*_2_ experiments used 10 CPMG delays from 8 ms to 240 ms, including two duplicates. *T*_1_ and *T*_2_ experiments had relaxation delays of 2.5 s and NOE experiments had a relaxation delay of 5 s. The spectra were analysed using CCPNmr Analysis 3.2.1 (Skinner et al. 2016) to obtain *R*_1_, *R*_2_ and NOE, and then by Modelfree 4.20 (Mandel et al. 1995). Measurements of viscosity and diffusion rate were made using a Fidabio fida 1 at 25 °C, 280 nm detection using intrinsic tryptophan fluorescence. Viscosity was determined from the fida elution time.

## Results

### Barnase plus betaine

**Fig. 1:**
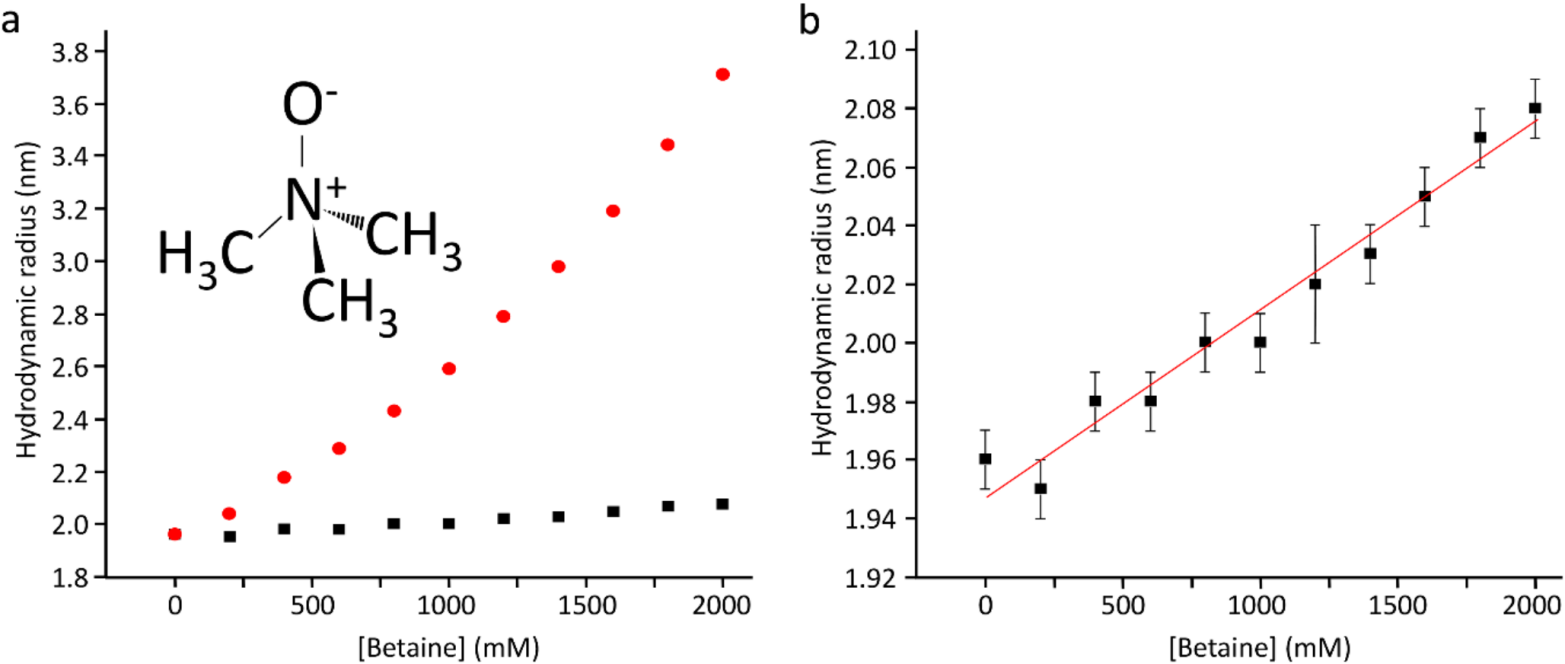
Hydrodynamic radius of barnase estimated using Fidabio, which measures the translational diffusion rate across a capillary, by detecting the profile as it passes a detector. (**a**) (red) Apparent hydrodynamic radius, calculated from the Stokes-Einstein equation; (black) True hydrodynamic radius corrected for solution viscosity as a function of betaine concentration. The chemical structure of betaine is shown. (**b**) True hydrodynamic radius (expansion of (a), with errors and fitting)

Barnase is an RNAse that catalyses a transesterification of RNA to make a 2’,3’ cyclic intermediate, which is then hydrolysed. It undergoes rather small conformational changes on binding of substrate. On titration of 1 M betaine into barnase, there was an increase in the viscosity of the solution, measured at just under 30% using Fida (Fig. 1a) (Jensen and Østergaard 2010). After correction for the viscosity, there is a linear increase in hydrodynamic radius of 0.64 ± 0.04 Å/M, consistent with replacement of some of the water in the hydration layer by betaine (Fig. 1b). Although betaine (Fig. 1, insert) is excluded from the protein surface, this does not mean that there is no betaine at the surface, merely that the concentration at the surface is lower than it would be in the absence of protein. The small increase in hydrodynamic radius is fully consistent with these expectations. There was a large ^1^H signal from the betaine at 3.25 ppm, which necessitated some reduction in the receiver gain in HSQC spectra. There was also an increased level of *t*_1_ noise and some bleed through of the betaine methyl signal into HSQC spectra, together with a small increase in pulse length. Despite these effects (which overall reduced the intensity of many signals by 20–30%), the quality of the HSQC spectra remained good even with 1 M betaine. We have previously reported an analysis of chemical shift changes in barnase on addition of betaine (Trevitt et al. 2024), which indicated that there is very little direct binding interaction between betaine and barnase. Similar observations were made here: no signals showed shift changes with betaine that were curved and could be convincingly fitted to give a dissociation constant, consistent with very weak binding. There were many chemical shift changes that were linear with betaine concentration, which we interpret (following Trevitt et al. 2024) as arising from interactions between betaine and water, in other words from a change in solvent structure or solvent fluctuations as betaine is added. As a further check on direct binding, saturation transfer difference (STD) spectra (Mayer and Meyer 1999) were collected of barnase in the presence of betaine, and showed no binding.

**Fig. 2:**
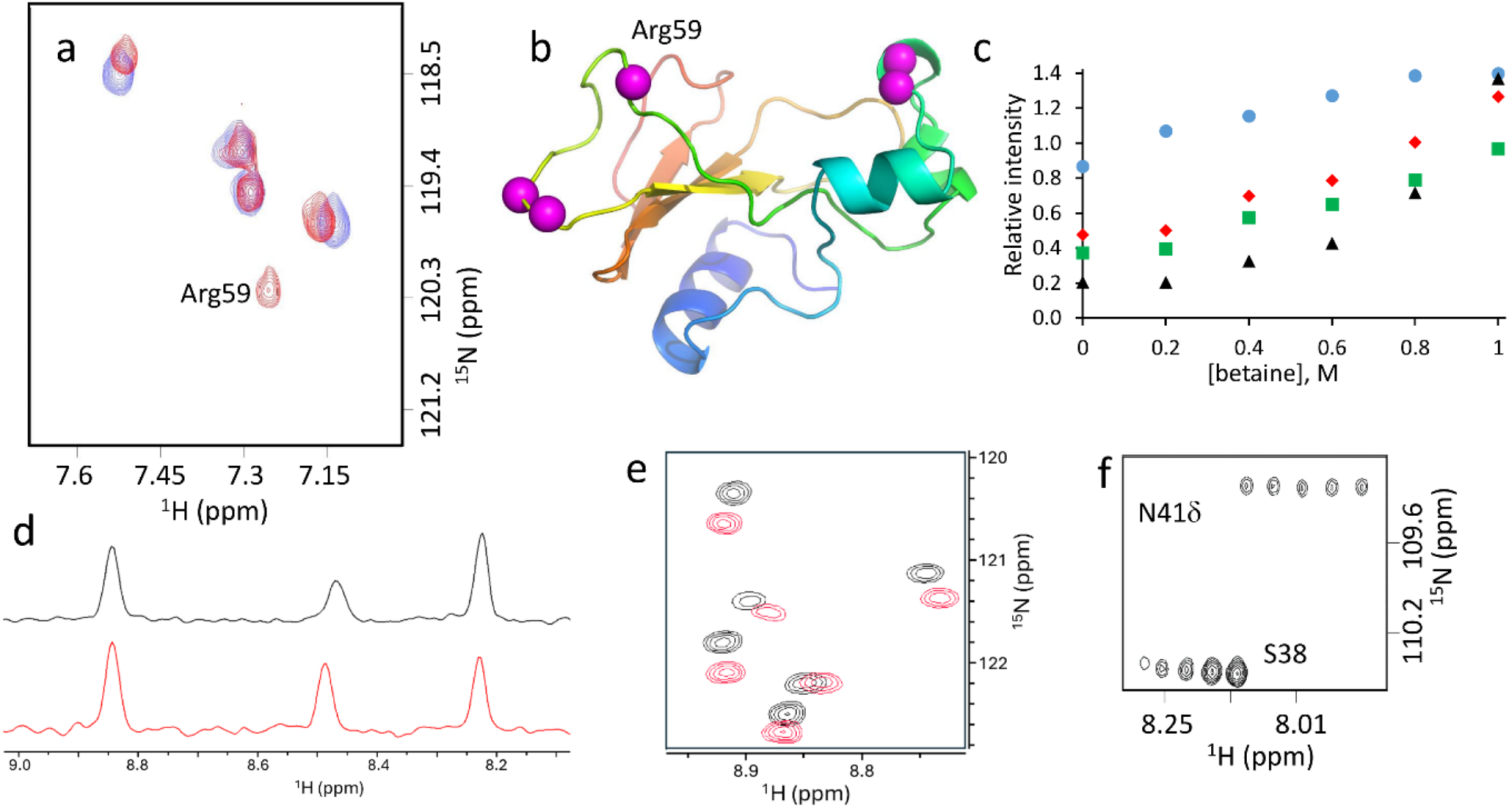
Increases in signal intensities in barnase on addition of betaine. (**a**) Part of the HSQC spectrum of barnase without betaine (blue) and with 1 M betaine (red). (**b**) Crystal structure of barnase (1a2p) showing the locations of the signals with the largest intensity increases. The active site is the shallow depression at top right. The backbone is coloured from blue at the N terminus to red at the C terminus. (**c**) Changes in intensity relative to an average of unaffected signals. Blue circles A37; green squares S38; black triangles S67; red diamonds: G68. (**d**) 1D slices at 122.9 ppm through HSQC spectra of barnase. Black without betaine, red plus 1 M betaine. (**e**) Part of the HSQC spectrum of barnase. Black without betaine, red plus 1 M betaine. Signal intensities were adjusted so that control signals (Asn/Gln sidechain amides) had the same intensity in both spectra. For clarity, the red spectrum has been shifted down. (**f**) A region of the HSQC spectrum containing signals for S38 and the sidechain of Asn41 (acting as an internal control). Spectra for 0, 0.4, 0.6, 0.8 and 1.0 M betaine are shown, keeping the intensity of the N41 signal at the same intensity, with each successive spectrum being displaced to the right

Most backbone amide signals in barnase can be observed, though there are some that are often absent or very weak. Most signal intensities reduce by approximately 25% on addition of 1 M betaine, due to a combination of increased viscosity and reduced receiver gain, the intensity change being linear with betaine concentration. After correction for this effect, roughly 80% of signals have intensities that do not change, with most of the others showing small linear reductions. There was only one absent signal that reappeared on addition of betaine, namely Arg59 (Fig. 2a). Arg59 is one of three backbone amides observed to form hydrogen bonds to the ligand in the crystal structure of barnase with d(CGAC) (Buckle and Fersht 1994), and sits in a loop that has µs/ms timescale dynamics in the d(CGAC) complex but not in the free state (Pandya et al. 2018) (Fig. 2b). In that study we were unable to characterise Arg59 itself because the signal could not be observed, in either the free or the bound state. Several other signals had significant increases in intensity on addition of betaine, namely Ala37, Ser38, Ser67 and Gly68 (Fig. 2c): these all sit at the tips of loops, and three of these (S38, S67 and G68) are absent from HSQC spectra in most of our previous studies (Pandya et al. 2018; Wilton et al. 2009). Thus, the common feature of all four is that they are typically weak or absent in HSQC spectra, and they are located near the ends of loops.

For most signals, there is no clear change in linewidth on addition of betaine (Fig. 2de), although overall there is a loss in signal-to-noise resulting from the addition of betaine. There is however a group of weak signals that is markedly more intense (Fig. 2cf). Considering that the increase in viscosity is approximately 25%, it is surprising that there is no obvious change in ^1^H linewidth. Possibly the window function has sharpened the signals enough to remove any obvious line broadening.

**Fig. 3:**
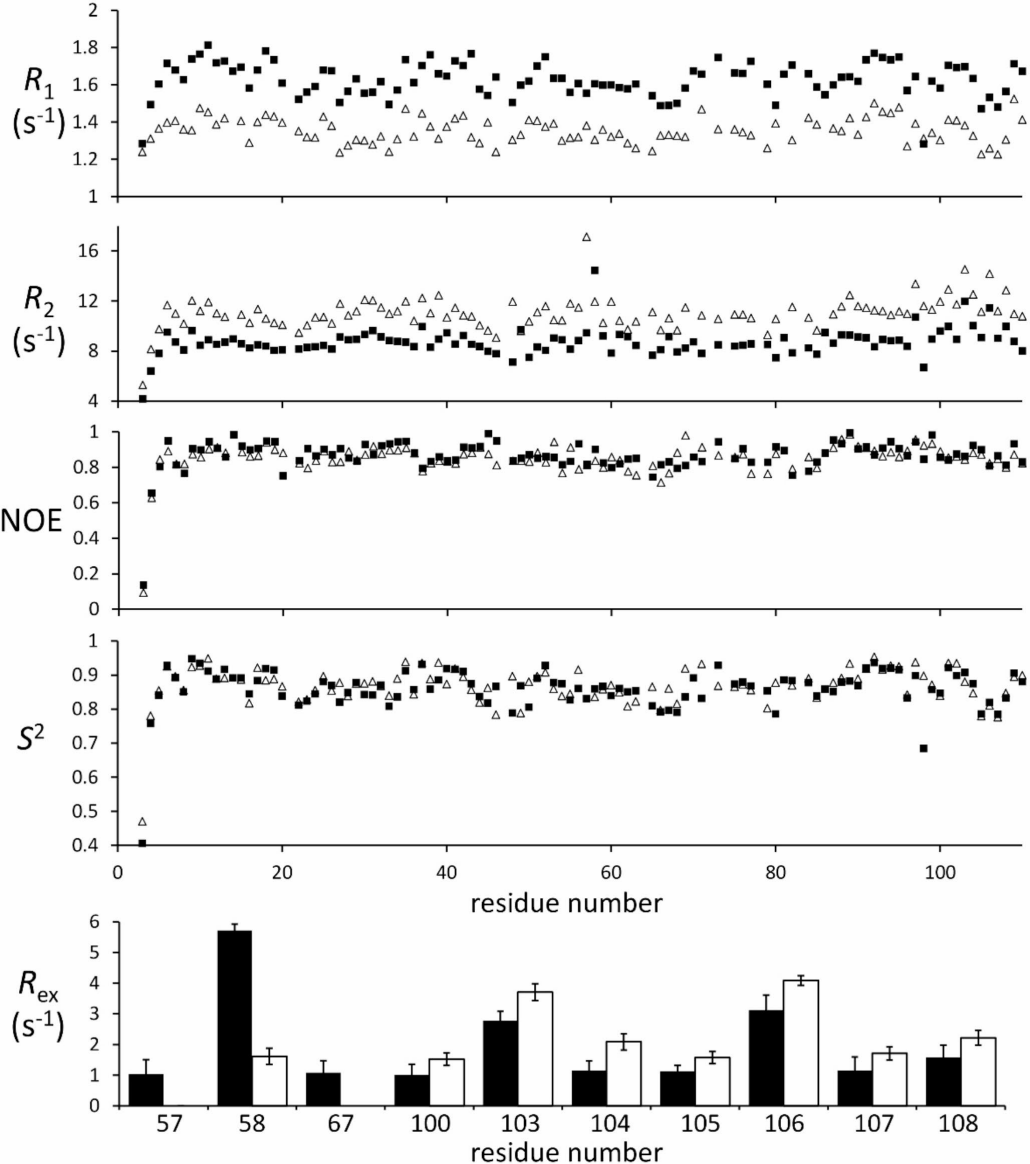
Relaxation parameters for barnase. Solid: barnase alone. Open: with 1 M betaine

The dynamics of barnase was probed using ^15^N relaxation. ^15^N *R*_1_, *R*_2_ and NOE were measured and analysed using Modelfree (Mandel et al. 1995) to derive order parameters. The results are shown in Fig. 3, and show, as reported previously (Sahu et al. 2000), that barnase has limited internal dynamics, with an average order parameter of 0.865, with only the N terminus and residue 98 having an order parameter below 0.7. There is nothing noteworthy about the order parameters of the five residues listed above or the loops that they sit in. Addition of 1 M betaine increased the average order parameter by a small but significant amount (to 0.873, *p* < 0.01, Student’s *t*-test), and the results indicated the expected increase in correlation time, from 6.64 to 8.53 ns. In barnase alone, most residues fitted to the simplest model of Modelfree, i.e. requiring just the order parameter, although 9 residues required the addition of a correlation time τ_e_ characterising slightly slower motions. Only 10 residues required an *R*_ex_ term, characteristic of motions on the µs/ms timescale. On addition of 1 M betaine, fewer residues required τ_e_ (only 3): in conjunction with the increased order parameters, this implies a more rigid protein at fast timescales. Some *R*_ex_ terms decreased, and some increased (Fig. 3e), implying no consistent change in motions on the µs/ms timescale.

**Fig. 4:**
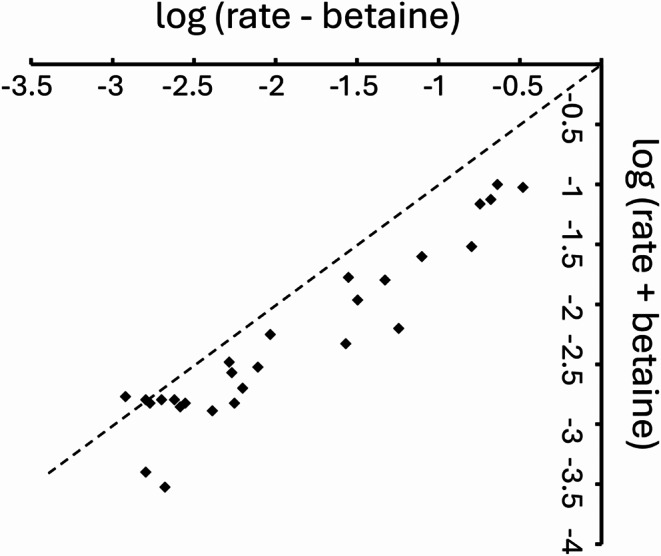
Amide proton exchange rates of slowly exchanging amides in the presence and absence of 1 M betaine. The values are given as log_10_(rate in hr^− 1^). The double log plot is used to emphasise that the slowing down of amide exchange in the presence of betaine is similar over a wide range of exchange rates, using a dashed line with gradient = 1 merely to guide the eye

Amide exchange rates were measured in the absence and presence of 1 M betaine. Exchange rates in 1 M betaine were slower by a factor of 2.8 ± 1.0 (Table 1). The exchange rate was slowed by roughly the same factor over a wide range of measured rates (Fig. 4). This suggests that the rare unfolding events that lead to amide exchange are less frequent (roughly by a factor of 3) in the presence of 1 M betaine.

**Table 1:** Amide exchange rates in Barnase (30 °C, pH 6.7). The residues listed are those for which confident rates were measured in both conditions

Residue	Exchange rate (hr^− 1^), no betaine	Exchange rate (hr^− 1^), 1 M betaine
11	0.057	0.0063
12	0.23	0.1
13	0.027	0.0047
14	0.0016	0.0015
15	0.0093	0.0056
17	0.18	0.0689
19	0.0024	0.0016
25	0.0016	0.0004
26	0.079	0.025
30	0.0026	0.0014
31	0.16	0.0304
33	0.032	0.0109
35	0.21	0.0751
45	0.33	0.0945
46	0.0078	0.0030
49	0.0052	0.0033
50	0.002	0.0016
51	0.0054	0.0027
56	0.0063	0.0020
75	0.0021	0.0003
76	0.0028	0.0015
87	0.047	0.016
88	0.0017	0.0015
89	0.0012	0.0017
95	0.028	0.0168
97	0.0016	0.0016
98	0.0041	0.0013
99	0.0056	0.0015

### Flap endonuclease plus betaine

Flap endonuclease (FEN) is approximately 650 residues long, with the endonuclease domain (studied here) containing 350 residues. It removes the single-stranded 5’ overhang on a DNA duplex, for which it has an active site containing divalent metal ions, as well as an arch formed from two helices, through which the single-stranded DNA strand passes (AlMalki et al. 2016). This arch is disordered in the absence of substrate and becomes ordered when the enzyme is bound to single stranded DNA (Balakrishnan and Bambara 2013). The protein used here is from the protozoan parasite *Plasmodium falciparum*.

**Fig. 5:**
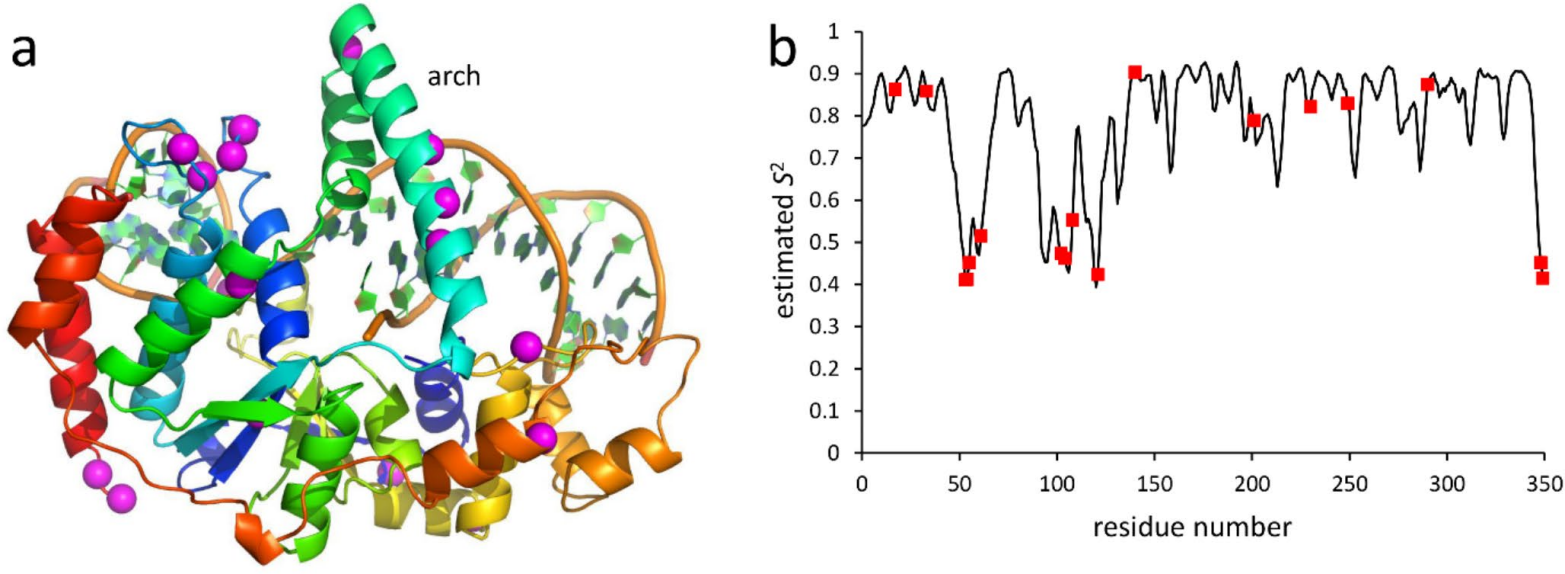
Flap endonuclease, indicating residues having increases in intensity. (a) Structure of FEN (AlphaFold2 prediction for *Plasmodium falciparum* FEN), including position of the DNA substrate, coloured from blue at the N terminus to red at the C terminus. Residues with intensity increases are highlighted in magenta. (b) Order parameter values estimated for FEN based on backbone chemical shifts and predicted by TALOS-N. Residues with intensity increases are highlighted in red. The arch is residues 40–60, and the region 90–120 with low order parameters is in contact with the DNA substrate

On addition of betaine to FEN, 17 backbone amide signals (out of a total of 299 assigned signals) showed increases in intensity. These signals are found all over the protein, including several from in and around the arch (Fig. 5a). Order parameters were estimated using TALOS (Shen and Bax 2013) based on backbone chemical shifts (Berjanskii and Wishart 2008), showing that many of the enhanced signals come from regions with low order parameters (Fig. 5b). As for barnase, STD experiments showed that there is no discernible binding of betaine to FEN.

## Discussion

There are several reports that addition of osmolytes to proteins increases the order parameter (Doan-Nguyen and Loria 2007; Lamosa et al. 2003; Pais et al. 2009), and leads to slower amide exchange (Foord and Leatherbarrow 1998; Jaravine et al. 2000; Kim et al. 2003; Lamosa et al. 2003; Pais et al. 2009; Qu and Bolen 2003; Wang et al. 1995), particularly of amides that already have slow exchange (i.e., from hydrogen bonded amides). However, to the best of our knowledge, this is the first report showing improvement in signal intensity. We therefore propose that osmolytes may be useful in improving the quality of HSQC spectra. The widespread observation, confirmed here, that osmolytes do not bind to the protein surface suggests that they do not have a major effect on protein structure or function: and indeed, it has often been observed that osmolytes have the effect of preserving or restoring protein function under stressful conditions such as high salt concentration (Yancey 2005).

Betaine is one of many possible osmolytes: we chose it here because it presented the least evidence of binding to the protein surface in our previous study (Trevitt et al. 2024). Addition of betaine up to 1 M can have adverse effects because it increases the viscosity, increases the *R*_2_ rate and decreases the *R*_1_ rate, but the main disadvantage is that it forced us to reduce the receiver gain. This problem could in principle be alleviated by using deuterated betaine. Addition beyond 1 M gave no obvious further benefit, and further exacerbated receiver overload, and is not recommended.

The order parameter measurements clearly indicate that betaine reduces rapid (ps/ns) motions in the protein. Betaine is known to strengthen hydrogen bonding within bulk solvent, and to increase long-range order and reduce fluctuations in bulk solvent (Bennion and Daggett 2004; Zou et al. 2002). This would be expected to decrease fluctuations in the protein co-solute, explaining the increase in order parameter. There is also a clear reduction in amide exchange rates, extending to the slowest exchanging amides, which have been shown to occur from a global unfolding event (Perrett et al. 1995). Large-scale protein fluctuations such as unfolding are widely agreed to be slaved to bulk solvent fluctuations (Frauenfelder et al. 2009; Qin et al. 2016). Thus, both the order parameter and the amide exchange results are consistent with a reduction in protein fluctuations linked to a reduction in the fluctuations in bulk solvent caused by betaine. The selective increase in intensity for a group of weak signals is suggested to result from this reduction in fluctuations, because the fluctuations lead to a loss in intensity due to chemical exchange. However, our results and those of others (Doan-Nguyen and Loria 2007; Lamosa et al. 2003) suggest that there is no clear effect on µs/ms motions, specifically the motions that produce *R*_ex_ terms. Although these motions ultimately derive their energy from thermal fluctuations in the solvent (Pandya et al. 2018), the linkage is still not fully understood and there would seem to be no simple relationship between solvent fluctuations and *R*_ex_ terms.

In summary, we have shown that addition of 1 M betaine markedly increases the intensity of several weak signals in the HSQC spectra of barnase and flap endonuclease, although it generally decreases the signal-to-noise by 25–30%. The mechanism is that betaine reduces fluctuations in bulk water, which in turn reduces fluctuations in the proteins. Adding betaine (or other osmolytes) thus does not improve intensity overall, but can be useful for enhancing some weak signals.

## Data Availability

Key data described here (barnase intensities as a function of betaine concentration, input T1, T2 and NOE data and ModelFree output files, amide exchange intensities as a function of exchange time) have been deposited with BioMagResBank as bmrbig104.
